# *Notes from the Field:* Cluster of Blastomycosis Among Neighborhood Residents — St. Croix County, Wisconsin, 2022

**DOI:** 10.15585/mmwr.mm7213a5

**Published:** 2023-03-31

**Authors:** Hannah E. Segaloff, Karen Wu, Summer Shaw, Elizabeth M. Klasen, Lori Peterson, Sue Lindberg, Samantha L. Williams, Andrew Wiese, Yvonne M. Bellay, Meredith Smith, Kelli Engen, Mitsuru Toda, Suzanne Gibbons-Burgener

**Affiliations:** ^1^Wisconsin Department of Health Services; ^2^Career Epidemiology Field Officer Program, CDC; ^3^Division of Foodborne, Waterborne, and Environmental Diseases, National Center for Emerging and Zoonotic Infectious Diseases, CDC; ^4^Council of State and Territorial Epidemiologists, Atlanta, Georgia; ^5^St. Croix County Public Health, New Richmond, Wisconsin; ^6^Bureau of Animal Disease Control, Department of Agriculture, Trade and Consumer Protection, Madison, Wisconsin; ^7^Countryside Veterinary Clinic, New Richmond, Wisconsin.

Blastomycosis, caused by the fungus *Blastomyces*, is a rare but potentially serious infection in humans and animals. *Blastomyces* is endemic in Wisconsin, which reports the highest incidence of *Blastomyces* infection in the country, with an estimated annual statewide incidence of 2.1 cases per 100,000 residents. Some high-incidence counties report 20–40 cases per 100,000 population (*1*,*2*). *Blastomyces* is also found in other midwestern, south-central, and southeastern states, and lives in moist, organic soils and decaying wood and leaves. Infections typically occur when *Blastomyces* spores are inhaled. *Blastomyces* infections do not spread between humans and animals through the air. Blastomycosis usually begins with mild respiratory symptoms, which often self-resolve, but can progress to a severe, and occasionally fatal, disease without antifungal treatment. In February 2022, a veterinarian in St. Croix County, Wisconsin, alerted the Wisconsin Department of Agriculture, Trade and Consumer Protection (DATCP) and the Wisconsin Department of Health Services (DHS) of four dogs with diagnoses of blastomycosis, all living within a 1-mile area. Review of surveillance data identified two human cases reported in the same area within 3 weeks of the canine cases. With 1–5 human cases reported annually, St. Croix County is not considered an area with hyperendemic transmission.

In response to this cluster, Wisconsin DHS and DATCP issued alerts to physicians and veterinarians in the surrounding counties, emphasizing the importance of timely diagnosis and treatment of blastomycosis. In Wisconsin, blastomycosis is reportable in humans but not in animals, and this alert encouraged local veterinarians to report cases potentially associated with this cluster. St. Croix County Public Health sent a letter to residents of the affected neighborhoods alerting them to the cluster and encouraging them to seek care if they had compatible symptoms. During January–March 2022, four persons and five dogs received a clinical diagnosis of blastomycosis. Two of the human cases received a diagnosis only after notification of the ongoing cluster. The five dogs with blastomycosis initially had mild to moderate symptoms: four experienced cough, difficulty breathing, lethargy, fever, and poor appetite. One dog had only a subcutaneous mass that contained *Blastomyces* yeast visible on microscopy. Urine antigen tests were positive for all infected dogs. Among the four persons with a clinical diagnosis of blastomycosis, all experienced cough, fever, and shortness of breath; symptom onset ranged from early October 2021 to early February 2022; two patients had presumptive laboratory evidence of infection, and two had confirmatory laboratory evidence.^†^ Two patients had severe disease and required hospitalization, including one adult patient who died. Antifungal medications are used to treat blastomycosis in humans and dogs and are often required for extended periods, depending on disease severity; all five canine and four human cases were treated with antifungal medications. Before January 2022, no blastomycosis cases had been reported in residents of this neighborhood during the preceding 10 years, although one dog reportedly died of blastomycosis during the previous year.

Environmental assessments identified a river and unpaved paths running through the neighborhoods under investigation. Construction in this neighborhood during the past decade might have dispersed *Blastomyces *spores. A more comprehensive investigation was launched to characterize potential environmental exposure sources in this community. Analysis of these data is ongoing.

Although blastomycosis is infrequently reported, clusters have occurred primarily among persons engaging in recreational activities along waterways or in areas with ongoing excavation (*3*–*5*). Clinicians should consider blastomycosis among patients with compatible symptoms who live in or have traveled to known areas of endemicity, especially among patients with respiratory symptoms that do not resolve with antibiotic treatment. Available diagnostic tests for blastomycosis include fungal culture, cytologic smear, histopathology, identification of *Blastomyces*-specific nucleic acids through polymerase chain reaction or DNA probe, antigen assay, or antibody detection by immunodiffusion or enzyme immunoassay. This investigation highlights the critical contribution of a multidisciplinary One Health^§^ approach in public health in which an astute veterinarian recognized and reported the canine cases leading to identification of the cluster.

## Footnotes

†
https://ndc.services.cdc.gov/case-definitions/blastomycosis-2020/

§
https://www.cdc.gov/onehealth/index.html

## References

[1] Benedict K, Gibbons-Burgener S, Kocharian A, . Blastomycosis surveillance in 5 states, United States, 1987–2018. Emerg Infect Dis 2021;27(4):999–1006. 10.1007/s12281-012-0110-1 33757624 PMC8007286

[2] CDC. Blastomycosis statistics. Atlanta, GA: US Department of Health and Human Services, CDC; 2022. Accessed October 17, 2022. https://www.cdc.gov/fungal/diseases/blastomycosis/statistics.html#one

[3] Baumgardner DJ, Burdick JS. An outbreak of human and canine blastomycosis. Rev Infect Dis 1991;13:898–905. 10.1093/clinids/13.5.898 1962106

[4] Klein BS, Vergeront JM, DiSalvo AF, Kaufman L, Davis JP. Two outbreaks of blastomycosis along rivers in Wisconsin. Isolation of *Blastomyces dermatitidis* from riverbank soil and evidence of its transmission along waterways. Am Rev Respir Dis 1987;136:1333–8. 10.1164/ajrccm/136.6.1333 3688635

[5] Cockerill FR III, Roberts GD, Rosenblatt JE, Utz JP, Utz DC. Epidemic of pulmonary blastomycosis (Namekagon fever) in Wisconsin canoeists. Chest 1984;86:688–92. 10.1378/chest.86.5.688 6488903

